# Consumers’ willingness to buy meat and seafood products close to the expiry date: an exploratory study from Denmark

**DOI:** 10.3389/fnut.2024.1371634

**Published:** 2024-03-12

**Authors:** Sujita Pandey, Amanda Bohl, Vittoria Favari, Pietro Mora, Sudikshya Phuyal, Eliška Sojková, Mausam Budhathoki, Marianne Thomsen

**Affiliations:** ^1^Department of Food Science, University of Copenhagen, Frederiksberg, Denmark; ^2^Institute of Aquaculture, University of Stirling, Stirling, United Kingdom

**Keywords:** consumer behaviour, expiry date, food waste, meat and seafood products, consumer attitudes

## Abstract

**Introduction:**

Meat- and seafood products close to their expiry date (MSPCED) are one of the significant contributors to the growing food waste. Therefore, this study aims to investigate consumers’ attitudes and willingness to buy MSPCED.

**Methods:**

An online questionnaire was used to collect data from 400 Danish consumers.

**Results and discussion:**

Three consumer segments were identified based on their willingness to buy MSPCED: 39.8% of the participants showed a high willingness to buy MSPCED close to their expiry date (“All High”), 34.5% were selective with a high willingness to buy meat close to their expiry date but not seafood (“High meat, low seafood”), while 25.7% showed a low willingness to buy MSPCED (“All Low”). Consumers’ willingness to buy MSPCED is influenced by the perceived quality of the products, food safety, social acceptability, and price. Consumers were willing to pay a higher price for minced beef close to the expiry date when compared to cod fillets, pork cuts, and chicken breasts. However, consumers were willing to buy cod fillets with the lowest discount percentage when compared to pork cuts, minced beef, and chicken breast. The findings suggest that price reduction and discount percentage can have varying effects in influencing willingness to pay for MSPCED. This study provides valuable insights, for food waste practitioners in the retail sector, to develop effective strategies for reducing food waste by influencing consumer willingness to buy and pay for perishable products like meat and seafood.

## Introduction

Global climate change severely threatens populations worldwide (1, 2). To date, only clean energy solutions have gained increasing attention, although around 26% of the overall greenhouse gas (GHG) emissions are attributed to the current food system (3, 4). According to the United Nations Environment Programme (UNEP), 8–10% of total GHG emissions are attributable to food loss and waste (FLW) (5). Considering that one-third of all food produced goes to waste, FLW prevention strategies become a crucial opportunity for significantly reducing the required food production and lowering GHG emissions (6). According to previous studies, approximately 32–61% of total FLW can be avoided through better coordination between the stakeholders of the food system (7–9). Halving FLW in the global supply chains would save 8% of the GHG emissions or 51 million tonnes of CO_2_ equivalents, along with an associated saving of 12% or 106,446 km^2^ of agricultural land use, 7% of water consumption (4.6 billion m^3^), and 14% of the energy [131 billion kWh; Osei-Owusu et al. (10)].

The largest meta-analysis of global food systems to date has shown significant differences in the GHG emissions of various food types (11). Generally, animal-based food production, particularly red meat products, contributes to at least twice as much total GHG emissions than their counterparts (3, 12). This shifted the world view on the loss and waste of meat and seafood products to recognise it as a complex and urgent concern that requires coordinated action from individual consumers to global policymakers. Researchers have argued that supplementing a reduction in emission-intensive animal-based food production and consumption by implementing effective strategies to prevent the loss and waste of meat products represents significant footprint savings (13–16).

At the global level, wastage of meat and seafood products amounts to 23% of meat and 35% of seafood products annually (14). Food supply chain loss and waste associated with European consumption are as follows: for meat, around 6% is lost at the primary, postharvest handling and storage and processing and manufacturing stages, while 17% is wasted at the retail and distribution, food service and households’ stages. For fish, the loss and waste percentages are 21 and 24% for fresh fish and 44 and 12% for processed fish (17). The most common reason for FLW at retail stores is that their expiry date has passed (18). While the expiry date provides guidelines, consumers are generally concerned regarding the food safety, nutritional and sensorial properties, even though assured by the manufacturer when stored in the recommended condition (19–21). So far several strategies have been proposed and implemented to reduce food wastage nearing its expiry date including Internet of Things (IoT) enabled technologies (22, 23), sales promotion (24, 25), packaging innovations (26, 27), and consumer communication (28, 29).

In Denmark, despite ambitious climate goals, the appetite for meat and seafood products is one of the highest in the world, with an estimated 52 kg *per capita* per year for meat and 22.1 kg *per capita* per year for seafood consumption (30). The GHG emissions, measured in CO2 equivalents per kilogramme, are 50 for lamb, 30 for beef, 10 for pork, 4 for chicken, and 6.5 for frozen fish (31). Furthermore, it has been estimated that 31% of Danish dinners contain beef or lamb (10), which is a critical notion as red meat products are known to have the highest environmental impact. In light of growing climate change awareness, more Danes have indicated an increasing willingness to reduce their intake of meat and seafood products (32). However, as much as it is consumed, meat and seafood are thrown away in Danish households, especially due to confusion regarding date labelling and uncertainty about shelf life (33, 34). The date labels are often misunderstood and consumers evaluate the quality, freshness and sensory characteristics of food solely based on the expiry labels (35, 36). The Danish food service sector has attempted to lower the price of meat and seafood products close to the expiry date (MSPCED) aiming to reduce food waste (37, 38). However, little is known about how Danish consumers perceive such incentives or how this affects their attitudes and willingness to buy. Hence, it is imperative to conduct research to evaluate consumers, attitudes and willingness to buy MSPCED. This knowledge can be invaluable for future management and mitigation efforts aimed at reducing meat and seafood product waste in the retail, food service sectors, and households.

Existing literature shows that several factors influence consumers’ willingness to buy perishable products close to expiry dates. These factors include food safety concerns, perceived nutritional quality, sensory perception, brand, and pricing (39–45). However, there is a lack of research investigating attitudes and behaviours towards MSPCED or the prices consumers are willing to pay for such products (46). This knowledge may be useful to the meat and seafood enterprise, retail, and food service sectors striving to decrease FLW. Therefore, the main objective of this study is to identify key factors influencing consumers attitudes and willingness to buy MSPCED. Additionally, the study categorises consumer segments according to their willingness to buy MSPCED providing insights into their attitudes and behaviour as well as their willingness to pay for such products. In this study, we are only referring to the MSPCED close to the expiry date and not the best-before date.

## Methods

### Data collection

Data was collected using an online questionnaire through SurveyXact platform. Snowball sampling was applied to collect the data, for instance, the participants were also asked if they could share the survey link among their social circles (47). Social media platforms, such as Facebook groups and LinkedIn targeting meat and seafood-eating consumers were used. Inclusion criteria to participate in the survey were the age range of 18–65 years old, consuming meat and seafood, and being willing to participate. A printed QR code for the survey was displayed in different spaces of the University of Copenhagen, visible to by-passers. The survey was open for responses from May 22nd until June 12th, 2022.

The questionnaire was first developed in English and was later translated into Danish to distribute in both languages. The purpose of having the questionnaire in two languages was to reach out as many participants as possible. The questionnaire was inspired by previous consumer studies on date labels of food products (48–50). The questionnaire was pilot-tested with 20 general consumers. The study obtained ethical approval from the Research Ethics Committee of Science and Health at the University of Copenhagen (Journal no.: 504–0364/22–5,000) and followed the Declaration of Helsinki.

The questionnaire consisted of four sections and 41 variables in total. In the first section, we asked about the participants’ socio-demographic characteristics, age, gender, education, income, region of residence, and the size of the city (see Table 1). The second section was about their willingness to buy MSPCED and their buying behaviour (see Table 2). We used a 7-point scale to measure how willing they were to buy, from “definitely not willing” to “definitely willing,” and a 5-point scale to measure how often they bought MSPCED, from “never” to “more than 4–5 times a month.” The third section had statements about their attitudes, such as whether they think MSPCED are still good quality, which they rated on a 7-point Likert scale from “strongly disagree” to “strongly agree” (see Tables 3, 4). The fourth part asked about how much they would be willing to pay for MSPCED (see Table 5).

**Table 1:** Sociodemographic characteristics of each segment and the total sample.

Clusters	High meat, low seafood	All low	All high	Total sample	*p*-value
Number of participants (n)	138	103	159	400	
Age (mean ± SD)	35.59 ± 14.17	34.99 ± 15.01	34.91 ± 12.92	35.16 ± 13.88	0.906^a^
Gender n (%)					**<0.001** ^**b**^
Male	49 (35.5)	45 (43.7)	56 (35.2)	163(40.8)	
Female	89 (64.5)	58 (56.3)	103 (64.8)	237(59.2)	
Education n (%)					0.492^c^
Primary	2 (1.5)	3 (2.9)	10 (6.3)	15(3.7)	
Secondary	23 (16.7)	15 (14.6)	22 (13.8)	60(15)	
Bachelor	95 (68.8)	73 (70.9)	113 (71.1)	281(70.3)	
Master/PhD	4 (2.9)	5 (4.8)	3 (1.9)	12(3)	
Other	14 (10.1)	7 (6.8)	11 (6.9)	32(8)	
Denmark n (%)					0.053^b^
Capital	60 (43.5)	60 (58.3)	74 (46.5)	194(48.5)	
Zealand	26 (18.8)	9 (8.7)	18 (11.3)	53 (13.3)	
Mid Jutland	19 (13.8)	20 (19.4)	34 (21.4)	73(18.2)	
North Jutland	14 (10.1)	3 (2.9)	13 (8.2)	30(7.5)	
South Denmark	19 (13.8)	11 (10.7)	20 (12.6)	50(12.5)	
City size n (%)					0.002^c^
<100,000	58 (42)	27 (26.2)	57 (35.9)	142(35.5)	
>100,000	69 (50)	51 (49.5)	84 (52.8)	204(51)	
Unknown	11 (8.0)	25 (24.3)	18 (11.3)	54(13.5)	
Income n (%)					0.308^c^
<10,000	42 (30.4)	33 (32.0)	57 (35.8)	132(33)	
10,001–20,000	39 (28.3)	30 (29.1)	47 (29.6)	116(29)	
20,001–30,000	29 (21)	14 (13.6)	34 (21.4)	77(19.2)	
30,001–40,000	9 (6.5)	8 (7.9)	3 (1.9)	20(5)	
>40,001	10 (7.3)	9 (8.7)	11 (6.9)	30(7.5)	
Prefer not to say	9 (6.5)	9 (8.7)	7 (4.4)	25(6.3)	

^a^ANOVA, ^b^Chi-square, ^c^Kruskal-Wallis H.

**Table 2:** Behaviour related to consuming MSPCED.

	High meat, low seafood	All low	All high	Total sample	*p*-value
Number of participants (n)	138	103	159	400	
Buy close to expiry date (%)					**<0.001** ^**b**^
Yes	122 (88.4)	51(49.5)	149(93.7)	322(80.5)	
No	16(11.6)	52(50.5)	10(6.3)	78(19.5)	
Frequency of purchase (%)					**<0.001** ^**c**^
Never	7(5.1)	25(24.3)	6(3.8)	38(9.5)	
1 time per month or less	37 (26.8)	44 (42.7)	33 (20.8)	114(28.5)	
2–3 times per month	50 (36.2)	25 (24.3)	44 (27.7)	119(29.7)	
4–5 times per month	28 (20.3)	7 (6.8)	43 (27)	78(19.5)	
More than 5 times per month	16 (11.6)	2 (1.9)	33 (20.7)	51(12.8)	
How many days before the expiry date are you willing to buy a meat product? (%)					<0.001^c^
Same day of expiry	61 (44.2)	13 (12.6)	100 (62.9)	174(43.5)	
1 day before	47 (34)	15 (14.6)	36 (22.6)	98(24.5)	
2–3 days before	23 (16.7)	44 (42.7)	18 (11.3)	85(21.2)	
4 or more days before	7 (5.1)	31 (30.1)	5 (3.2)	43(10.8)	
Best possible use of MSPCED product^M^					
I will consume them immediately (%)	91 (65.9)	57 (55.3)	97 (61)	245(61.3)	0.247^b^
I will consume them as long as they do not pass the expiry date (%)	48 (34.8)	36 (35)	62 (39)	146(36.5)	0.702^b^
I will freeze them before the expiry date (%)	82 (59.4)	43 (41.7)	114 (71.7)	239(59.8)	**<0.001** ^**b**^
I will throw them away if they do not smell nice (%)	103 (74.6)	61 (59.2)	108 (67.9)	272(68)	**0.040** ^**b**^
I will throw them away if they do not look nice (%)	51 (37)	42 (40.8)	61 (38.4)	154(38.5)	0.833^b^
I will throw them away if they do not taste nice (%)	68 (49.3)	36 (35)	74 (46.5)	178(44.5)	0.069^b^
I will just throw them away (%)	3 (2.2)	6 (5.8)	-	9(2.3)	**0.004** ^**d**^

^a^ANOVA, ^b^Chi-square, ^c^Kruskal-Wallis H, ^d^Fisher’s exact, ^M^Multiple-response options.

**Table 3:** Attitudes toward MSPCED.

Attitude statement (AS)	High meat, low seafood median(IQR)	All low median(IQR)	All high median(IQR)	Total sample median(IQR)	*p*-value
AS1: I’m afraid I will get sick if I eat meat and seafood close to its expiry date. (R)	5(3)	4(3)	6(3)	5(3)	<0.001
AS2: I’m afraid I will not have time to prepare the meat and seafood product close to the expiry date. (R)	4(3)	3(3)	5(3)	4(3)	<0.001
AS3: I think it is unnecessary to buy meat and seafood close to the expiry date. (R)	5(3)	4(2)	5(3)	5(3)	0.065
AS4: I think buying meat and seafood close to the expiry date is reducing food waste.	6(1)	6(1)	6(1)	6(1)	<0.001
AS5: I think buying meat and seafood close to the expiry date is unappealing. (R)	6(3)	4(3)	6(2)	6(3)	<0.001
AS6: I think meat and seafood close to the expiry date does not taste as good as a product with a longer expiry date. (R)	6(2)	4(3)	6(3)	6(3)	<0.001
AS7: I think meat and seafood close to the expiry date does not have a good quality compared to a fresh product. (R)	5(3)	4(3)	6(3)	5(3)	<0.001
AS8: I think meat and seafood close to the expiry date is not as healthy as a product with a longer expiry date. (R)	6(2)	5(2)	6(1)	6(2)	<0.001
AS9: I always look for the expiry labels when I buy meat and seafood products.	6(2)	6(2)	6(2)	6(2)	0.276
AS10: I think the discounted price is very important when I buy meat and seafood products close to the expiry date.	7(1)	6(1)	6(2)	6(2)	<0.001
AS11: I think others would look down on me if I buy products close to the expiry date. (R)	6(2)	6(3)	7(1)	6(2)	0.007

R, Reverse scale, attitudes were measured on a 7-point Likert scale ranging from “Strongly disagree” to “Strongly agree,” IQR, Interquartile range.

**Table 4:** Likelihood of belonging to segment based on attitudes toward MSPCED.

Attitude statement (AS)	High meat, low seafood		All low		All high	
	OR	CI	OR	CI	OR	CI
AS1: I’m afraid I will get sick if I eat meat and seafood close to its expiry date. (R)	0.880	0.759–1.037	0.939	0.778–1.133	**1.185**	**1.010–1.391**
AS2: I’m afraid I will not have time to prepare the meat and seafood product close to the expiry date. (R)	1.009	0.889–1.144	0.924	0.793–1.077	1.021	0.898–1.549
AS3: I think it is unnecessary to buy meat and seafood close to the expiry date. (R)	0.929	0.816–1.057	**1.199**	**1.043–1.378**	0.926	0.816–1.049
AS4: I think buying meat and seafood close to the expiry date is reducing food waste.	1.071	0.870–1.318	**0.734**	**0.589–0.914**	**1.247**	**1.003–1.549**
AS5: I think buying meat and seafood close to the expiry date is unappealing. (R)	1.141	0.952–1.368	**0.778**	**0.637–0.950**	1.097	0.908–1.325
AS6: I think meat and seafood close to the expiry date does not taste as good as a product with a longer expiry date. (R)	1.121	0.904–1.391	0.943	0.734–1.167	0.913	0.729–1.143
AS7: I think meat and seafood close to the expiry date does not have a good quality compared to a fresh product. (R)	**0.795**	**0.663–0.954**	0.932	0.745–1.167	1.311	1.085–1.585
AS8: I think meat and seafood close to the expiry date is not as healthy as a product with a longer expiry date. (R)	1.196	0.962–1.488	0.799	0.635–1.006	1.048	0.834–1.317
AS9: I always look for the expiry labels when I buy meat and seafood products.	0.980	0.861–1.115	0.933	0.807–1.080	1.074	0.949–1.215
AS10: I think the discounted price is very important when I buy meat and seafood products close to the expiry date.	**1.223**	**1.032–1.450**	**0.819**	**0.687–0.975**	0.961	0.823–1.122
AS11: I think others would look down on me if I buy products close to the expiry date. (R)	0.923	0.782–1.089	1.029	0.844–1.254	1.078	0.906–1.281

The bold numbers represented a significantly higher likelihood of being in the segment when agreeing with the statement per increment on the Likert scale. OR, Odds ratio, CI, Confidence Interval.

**Table 5:** Average price and discount percentage consumers are willing to pay for MSPCED.

	High meat, low seafood		All low		All high		Total sample		*p*-value^a^
	Mean	SD	Mean	SD	mean	SD	Mean	SD	
1 kg of minced beef priced at 100 DKK	51.5	19.1	34.6	29.2	50.2	24.4	46.6	25.1	<0.001
1 kg of pork cut priced at 58 DKK	26.2	14.5	13.9	15.4	26.9	15.4	23.3	16.0	<0.001
1 kg of chicken breast priced at 104.4 DKK	46.6	25.0	41.8	27.6	53.2	24.8	47.9	25.9	0.002
1 kg of Cod fillets priced at 177 DKK	51.7	49.0	47.9	53.6	87.9	46.3	65.1	52.4	<0.001
Discount Percentage for 1 kg of minced beef	41.4	21.1	31.5	30.4	38.4	22.1	37.7	23.2	0.005
Discount Percentage for 1 kg of pork cut	38.1	24.1	31.5	30.4	37.5	22.7	36.2	25.4	0.097
Discount Percentage for 1 kg of chicken breast	38.0	23.5	37.7	26.5	40	20.7	38.3	23.3	0.896
Discount Percentage for 1 kg of Cod fillets	30.9	29.9	30	31.7	39.7	23.3	34.2	28.3	0.006

a One-way ANOVA test.

### Data analysis

After the screening, responses from 400 consumers were deemed acceptable for the subsequent data analysis using SPSS v29 (51). Categorical data were presented as frequency and proportions, while continuous data were described with means and standard deviations.

Firstly, the K-means algorithm was used to segment consumer based on their willingness to buy MSPCED including beef, pork, poultry, cured meats/cold cuts/salami, fish, and shellfish. The K-means is a widely used and validated method for market segmentation, which utilises a machine learning algorithm to associate similar data points and understand the underlying patterns presented (52). Further, a gap statistic was applied in R studio (53) to verify the resulting three clusters solution, “All Low,” “High meat, low seafood,” and “All High.” The difference between the three consumer segments was determined through analysis of variance (ANOVA), Kruskal-Wallis H, Chi-square, and Fisher’s exact tests depending on the nature of data and types of variables (54).

Logistic regression was employed to assess the likelihood of belonging to the clusters based on a list of attitudinal statements. The dependent variables were segment membership, whereas the independent variables were 11 attitudinal questions covering different dimensions of buying meats close to the expiry date, such as perceived safety and healthiness, product desirability, social acceptance etc. The model also controlled for sociodemographic and behavioural variables, which showed significant differences across the segments. The function form of the logistic regression model used is represented as follows:


$$Z_{i}=\ln\left[\frac{P_{i}}{1-P_{i}}\right]=\beta_{0}+\beta_{1}H_{i}+\beta_{2}Q_{i}+\beta_{3}Y_{i}+\epsilon_{i}$$

where $Z_{i}$ is a log odds, ln denotes the natural logarithm, $\beta_{0}$ is constant, $\beta_{1}$, $\beta_{2}$ and $\beta_{3}$ are vectors of coefficients associated with variables $H_{i}$, $Q_{i}$ and $Y_{i}$ respectively. $\epsilon_{i}$ is an error term. The coefficient calculates a change in log odds of the dependent variable, not the change in the variable itself. Thus, interpreting a logit by converting it to odds ratio using the exponential function is the most common way to interpret relationships (55). The functional form of odds ratio is represented as follows:


$$\text{Odds ratio}=e^{\beta_{0}+\beta_{1}H_{i}+\beta_{2}Q_{i}+\beta_{3}Y_{i}+\epsilon_{i.}}$$

Here, the odds ratio is simply the ratio of the probability that the consumers belong to the clusters based on a list of attitudinal statements and higher odd ratios signify that consumers who agree with the statements are likelier to belong to that segment. Results are presented as odd ratios with associated *p*-values and confidence intervals. Further, ratio and proportion were used to simplify the explanation of the willingness to pay for MSPCED. Further, the difference in willingness to pay between consumer segments was determined by one-way ANOVA tests as it is commonly used to analyse the effects of a single categorical independent (with three or more levels) on a continuous dependent variable (54). *p*-values less than 0.05 were considered significant and are presented in bold in the results section.

## Results

Three consumer segments were identified based on their willingness to buy MSPCED: 39.8% of the participants showed a high willingness to buy MSPCED (“All High”), 34.5% were selective with a high willingness to buy meat close to their expiry date but not seafood (“High meat, low seafood”), while 25.7% showed a low willingness to buy MSPCED (“All Low”).

Table 1 presents the sociodemographic characteristics of each segment and the total sample. The segments are similar for most sociodemographic variables except for gender and city size. The average age of the participants across segments is between 34 and 36. Roughly half of the participants live in the capital region of Denmark and the other half in a city with at least 100.000 inhabitants. The participants are dominated by females (59.2%). Moreover, a majority of the participants (70.3%) have achieved tertiary education, and the majority (52%) have an income of less than 20,000 Danish krone (DKK) per month.

Table 2 presents the consumers’ behaviour related to consuming MSPCED. The results indicate that the majority of the participants (80.5%) had previously bought MSPCED and 58.2% of the participants purchase MSPCED 1–3 times per month. Most of the participants are willing to buy MSPCED on the same date of expiry and freeze them for later use. The results indicated that there is a significant difference between consumer segments concerning the behaviour of consuming MSPCED. For instance, most of the consumers from the “All High” segment were willing to buy MSPCED that expire on the same day or 1 day before expiry, while most of the “All Low” consumers were willing to buy such products with more than 4 days left before reaching the last expiry date.

Table 3 presents the median and interquartile range of 11 attitudinal statements to buying MSPCED. The results indicated that there is a significant difference in attitudinal statements between the consumer segments except for statement 3 (AS3) and statement 9 (AS9). All three segments consider noticing the expiration date when buying meat and seafood products. Further, the “High meat, low seafood” segment perceived that discounted price is important for buying MSPCED, while the “All High” segment considered MSPCED to have good quality.

Furthermore, none of the segments perceives buying MSPCED as socially disqualifying, and all segments recognise buying MSPCED as reducing food waste. Similarly, the consumers in the “All Low” segment find buying MSPCED less appealing than the other two segments. In conclusion, the three groups share a common perception of MSPCED; the more favourable “All High” and “High meat, low seafood” segments reveal more positive attitudes than the “All Low” segments.

Table 4 shows the result of the likelihood of belonging to the segment based on attitudes towards eating MSPCED. Consumers who are confident that they will not get sick after consuming MSPCED and buying such products to reduce food waste belong to the “All High” segment. Consumers belonging to the “High meat, low seafood” segment considered the discounted price important when buying MSPCED. The “All Low” consumer segments perceived buying MSPCED as necessary.

Table 5 shows the average price and discount percentage consumers are willing to pay for minced beef, pork cut, chicken breast and cod fillets close to the expiry date. The result indicated that there is a significant difference in willingness to pay for these products across the three consumer segments, except for the discount percentage of 1 kg of chicken breast. With reference to the 100 DKK / kg original price, the willingness to pay showed a slightly increasing trend ranging from about 54 to 63% reduction in the price, on average 37 DKK for cod fillets, 46 DKK for minced beef, DKK 40 for pork cuts, and 46 DKK for chicken breast. The “All High” segment was willing to pay higher prices for pork cuts, chicken breast and cod fillets, while the “High meat and low seafood” segment was willing to pay higher prices for minced beef. Further, consumers belonging to the “High meat, low seafood” segment were likely to purchase minced beef and pork cuts when higher discount percentages were given. While the “All High” segments were more likely to purchase chicken breast and cod fillets when higher discount percentages were given.

## Discussion

This study aims to investigate consumers, attitudes and willingness to buy MSPCED. The study identified three different consumer segments based on willingness to buy MSPCED: (1) “All High,” (2) “All Low” and (3) “High meat and low seafood.” Further, attitudes, behaviour and willingness to pay for MSPCED between the three consumer segments were explored. The results indicated that consumers’ buying behaviour towards the MSPCED is determined by their perceived quality, food safety, social acceptability, and price.

Perceived quality is regarded as a barrier to buying MSPCED and the finding from this study indicated that consumer segments perceived the quality of MSPCED differently. Previous studies have identified that the perception of food quality can be ambivalent. For example, food products with a short shelf-life may be perceived as high quality in terms of freshness, but at the same time, they can be perceived as low quality because the product may turn bad quickly (56). Consumers perceived that MSPCED are of lower quality only to a moderate extent, where the “All Low” segment remains the most sceptical regarding its quality, taste, and appeal. The finding aligns with previous studies that show how the label “close to expiry date” can affect consumers’ perception of the products’ quality negatively (57, 58). The findings from this study indicated that taste, smell, and look were assessed more closely to evaluate the quality of MSPCED, which may also indicate an increased risk of elevated food waste rates at the household level. A recent study found that consumers mainly rely on expiration dates and their senses to determine the freshness of meat and seafood products (35). Further, older consumers (55 years and above) were more likely to use their sensory skills to determine the quality and safety of a product rather than check expiry dates (59, 60). Thus, it is recommended to educate consumers through demonstration and experience to improve knowledge and trust in the expiration label and that smart labels could provide external validation in terms of the quality of MSPCED (35).

Fear of inadequate food safety is another crucial factor for discarding MSPCED and acts as a barrier to buying such products. The “All Low” segment, in particular, expressed fear about purchasing MSPCED, perceiving a risk of illness from consuming them. Additionally, they perceived MSPCED as less healthy compared to the “All High” and “High meat, low seafood” segments. Health is an abstract dimension commonly linked to subjective perceptions of nutritional and food safety in food products close to expiry (56). Further, the health risk is essential for consumers in determining consumption of food products close to the expiry date (61). Therefore, consumers use the expiration label, especially for highly perishable products, like meat (62) and seafood (63) to determine the potential health risk as a criterion for purchasing (64). Further, food safety concerns about meat and seafood products have become more important following the COVID-19 pandemic (65) and consumers seem to be extra cautious regarding such products. Information and knowledge are connected to behavioural aspects of consumers’ handling of MSPCED and have previously been of interest to the Danish Food Ministry and the Consumer Council, which has been using informational campaigns to promote information and knowledge about food safety issues (56). Apart from providing information and knowledge, novel packaging solutions targeting spoilage mitigation and smart sensors for dynamic shelf life labelling may support consumers’ preventive measures to mitigate food spoilage (66, 67).

Regarding the acceptability of MSPCED and consumers’ perceptions of food waste, all three segments recognise that buying MSPCED helps prevent food waste. Although the “All High” and “High meat, low seafood” segments tend to purchase MSPCED more frequently, they are also more likely to discard or refrain from consuming the product if it exhibits suspicious or unpleasant odours or colours. Previous research shows consumers often feel morally obligated to reduce food waste (46). Regarding the willingness to buy MSPCED, increasing consumer awareness on issues regarding food waste may increase consumers’ moral satisfaction in buying near-expired food (68). The research further highlights that using food waste avoidance messages that signal to buy near-expired food is a pro-environmental behaviour that can further increase consumers’ moral satisfaction in buying near-expired food.

Despite consumers’ awareness and recognition of food waste prevention in paying for MSPCED, pro-environmental behaviours do not always extend from supermarkets to household situations. Some research shows that according to moral licence, people who initially behave morally by paying for MSPCED may later engage in unethical behaviours (68, 69). In this regard, knowledge of managing expiration dates and reducing food waste may become more critical in the future. Support in the form of policy incentives could make this consumer behaviour more frequent as there is already an underlying positive perception about consuming products close to the expiration date (46).

The results from this study indicated that the price and discount percentages are important factors influencing consumers’ willingness to buy MSPCED. Depending on the product type, consumer willingness to pay for MSPCED differs. Further, price reduction and discount percentage can have varying effects in influencing willingness to pay for MSPCED. In line with this finding, price is considered the most crucial factor affecting consumer buying decisions (70–72) and is still the main barrier to consumers’ willingness to buy products close to the expiry date (71). In Denmark, a recent initiative by DanChurchAid focused exclusively on surplus goods, being the first initiative of its kind offering food products that regular supermarkets can no longer sell for reasons such as overdue ‘best before’ dates or damaged packaging at 30–50% below market prices (73). However, discounts are not the only way to promote sales as a recent study has shown that a message about food waste avoidance can suffice to increase consumers’ willingness to buy food close to the expiry date without recurring to lower prices (68). Thus, food close to expiry exclusively as cheap and arbitrarily discounted products does not always prompt consumers to buy them (74). However, it is notable that all three groups of consumers valued discounted prices and discounted percentages when buying MSPCED. Therefore, this study confirms that discounts keep playing a vital role in the willingness to buy perishable products approaching the expiry date. In Denmark, discounts remain a popular strategy (75), and food waste practitioners are interested in finding what price can influence willingness to buy MSPCED and maximise economic returns. Further, consumers do not perceive price promotions uniformly, so a one-size-fits-all price promotion may not be as effective in promoting prices as more nuanced approaches (74). This study did not address additional promotion efforts, including money-back guarantees or explicit instructions for cooking or storing a particular product (74). Thus, it is recommended to implement a dynamic pricing strategy by considering different factors such as the initial inventory age profile or the sensitivity of demand to the product age for reducing perishable food waste at retailers (76, 77).

The result from this study indicated that chicken breast retained its original price more than minced beef, pork cuts and cod fillets, but unless a higher discount percentage was provided consumers were less willing to buy them. Further, cod fillets lost most of their original value, but consumers were willing to buy them with less discount percentage. In general, seafood is expensive when compared to other meat products and consumers value its freshness, taste, and health and nutrition profiles (78–80). Further, seafood is generally perceived with higher food safety concerns than meat (81, 82). The greater the risks associated with a product, the more frequently consumers check expiration dates, resulting in decreased willingness to pay (50). This might explain why cod fillets close to the expiry date lose most of their value compared to other meat products. The findings are in line with previous studies that indicated that price change presented in discount percentages or monetary amounts stimulates consumers’ perceptions and its effect depends on the products’ regular price (83, 84). Thus, it is recommended that retailers test whether discount percentage, monetary amount and/or both optimise the sales of MSPCED while maximising the profit. Further, a study has found that combining discounting with dynamic shelf life strategies seems more effective in reducing food waste at the retail level (85).

A study from Collart and Interis (46) suggests that the willingness to buy MSPCED increases if purchased in frozen form. Another study described that the willingness to purchase the product could increase if the product were divided into single-packed portions. This study has not investigated this aspect, but consumers were freezing MSPCED for later use, especially among consumers from the “All High” and “High meat, low seafood” segments. While freezing one part and consuming another immediately, MSPCED product reduces potential waste, prolong the products’ shelf-life, and eliminate the need to consume them immediately (75). The findings also suggested that the timing of consuming MSPCED is a factor to consider when purchasing such products. Across all segments, more than half of consumers declared to consume MSPCED immediately, with even higher percentages for consumers in both “All High” and “High meat, low seafood” segments that seem to affect consumers’ willingness to buy MSPCED.

### Strengths and limitations of the study

The sample size of 400 consumers supports the appropriateness of online survey research (86). This study focuses on the attitudes and willingness to buy MSPCED, which has often been neglected. The results show that knowledge about the importance of how discount prices are communicated with the MSPCED to influence willingness to buy MSPCED could be relevant for future studies.

There are several limitations of this study. The term “close to expiry date” was left to the consumers’ interpretations to limit biassed answers. However, it has been estimated that 68.8% of Danish consumers correctly interpret the meaning of the “expiry date” label (56). The absence of questions on dietary preferences may have limited the collection of relevant data for explaining the characteristics of the segments. The snowball method was used as a sampling strategy, so there is a higher likelihood of biassed results (87). Further, participants were recruited through social media, which might have resulted in self-selection bias (88) and verification of information on the Web remains more difficult than in a face-to-face survey. However, studies have indicated that social media can be the best recruitment method for observational studies (89). We have only included four MSPCED for determining willingness to pay, which might have limited the inclusion of consumers’ preferred meat and seafood products. Further, individual taste preferences might also have influenced the results. For instance, in a situation where someone who eats meat, but does not eat seafood, or likes to eat seafood, but does not eat meat, then his/her willingness to buy MSPCED is likely to be different from that of an individual who likes both meat and seafood. Lastly, a high share of low-income (about 62%) and highly educated (about 73%) respondents in our sample, represents a bias in the representativeness of the sample.

## Conclusion

The present study identified three consumer segments based on their willingness to buy MSPCED, the “All Low” (25.7% of the participants), the “All High” (39.8%), and the “High meat, low seafood” (34.5%). Despite consumers’ awareness and recognition of food waste prevention in buying MSPCED, pro-environmental behaviours might not necessarily extend from supermarkets to households, as several consumers throw MSPCED after purchase due to (perceived or real) product spoilage. In this regard, knowledge of managing the MSPCED and understanding the attitude-intention-behavioural gap in reducing food waste may become a critical parameter for reducing food waste at the household level. Future studies might investigate this aspect and immediate consumption and portion size freezing at home could have provided us with a better insight into the knowledge and awareness among consumer segments regarding food waste prevention. Lastly, policymakers should implement regulations that enable flexible pricing for meat and seafood products close to the expiry date, enabling retailers to provide discounts without facing legal limitations. Retailers, in turn, should implement dynamic pricing strategies and cross-promotional campaigns to maximise sales of these products, while aiming to reduce food waste.

## Data availability statement

The raw data supporting the conclusions of this article will be made available by the authors, without undue reservation.

## Ethics statement

The studies involving humans were approved by Research Ethics Committee of Science and Health at the University of Copenhagen. The studies were conducted in accordance with the local legislation and institutional requirements. The participants provided their written informed consent to participate in this study.

## Author contributions

SPa: Conceptualization, Data curation, Formal analysis, Investigation, Methodology, Software, Validation, Visualization, Writing – original draft, Writing – review & editing. AB: Conceptualization, Data curation, Investigation, Methodology, Writing – original draft. VF: Conceptualization, Data curation, Investigation, Methodology, Writing – original draft. PM: Conceptualization, Data curation, Investigation, Methodology, Writing – original draft, Formal analysis. SPh: Conceptualization, Data curation, Investigation, Methodology, Writing – original draft. ES: Conceptualization, Data curation, Investigation, Methodology, Writing – original draft. MB: Formal analysis, Methodology, Software, Supervision, Validation, Visualization, Writing – review & editing. MT: Conceptualization, Funding acquisition, Methodology, Project administration, Resources, Supervision, Validation, Visualization, Writing – review & editing.
